# Molecular dynamics study on protein–water interplay in the mechanogating of the bacterial mechanosensitive channel MscL

**DOI:** 10.1007/s00249-015-1065-2

**Published:** 2015-08-02

**Authors:** Yasuyuki Sawada, Masahiro Sokabe

**Affiliations:** Department of Physiology, Nagoya University Graduate School of Medicine, 65 Tsurumai-cho, Showa-ku, Nagoya, 466-8550 Japan; Mechanobiology Laboratory, Nagoya University Graduate School of Medicine, 65 Tsurumai-cho, Showa-ku, Nagoya, 466-8550 Japan

**Keywords:** Mechano-gating, MscL, Molecular dynamics simulations, Vapor lock, Hydration, Hydrogen bond

## Abstract

One of the goals of mechanosensitive channel (MSC) studies is to understand the underlying molecular and biophysical mechanisms of the mechano-gating process from force sensing to gate opening. We focus on the latter process and investigate the role of water in the bacterial MSC MscL, which is activated by membrane tension. We analyze the interplay between water and the gate-constituting amino acids, Leu19–Gly26, through molecular dynamics simulations. To highlight the role of water, specifically hydration of the gate, in MscL gating, we restrain lateral movements of the water molecules along the water–vapor interfaces at the top and bottom of the vapor bubble, plugging the closed gate. The gating behaviors in this model and the normal MscL model, in which water movements are unrestrained, are compared. In the normal model, increased membrane tension breaks the hydrogen bond between Leu19 and Val 23 of the inner helix, exposing the backbone carbonyl oxygen of Leu19 to the water-accessible lumen side of the gate. Associated with this activity, water comes to access the vapor region and stably interacts with the carbonyl oxygen to induce a dewetting to wetting transition that facilitates gate expansion toward channel opening. By contrast, in the water-restrained model, carbonyl oxygen is also exposed, but no further conformational changes occur at the gate. This suggests that gate opening relies on a conformational change initiated by wetting. The penetrated water weakens the hydrophobic interaction between neighboring transmembrane inner helices called the “hydrophobic lock” by wedging into the space between their interacting portions.

## Introduction

Mechanosensitive channels (MSCs) are major cell mechanosensors that support physiology and life processes in organisms (Levina et al. 1999; Batiza et al. 2002; Kung 2005). MSCs are implicated in physiological functions such as hearing and touch sensing in animals as well as osmoregulation in bacteria (Martinac et al. 1987; Sukharev et al. 1993; Booth and Louis 1999; Hamill and Martinac 2001). MSCs were first reported in the 1980s (Hamill 1983; Guharay and Sachs 1984); however, the crystal structures of MSCs have beeen resolved only for a couple of bacterial MSCs: mechanosensitive channel large conductance (MscL) (Chang et al. 1998; Steinbacher et al. 2007) and mechanosensitive channel small conductance (MscS) (Bass et al. 2002).

MscL is a homopentameric structure whose subunit comprises two transmembrane (TM1 and TM2) helices connected via a periplasmic flexible loop and N-/C-terminal domains located at the cytoplasmic side. The closed-state structure of MscL from *Mycobacterium tuberculosis* was first determined by Chang et al. (1998) and refined by Steinbacher et al. (2007). Five TM1s line the ion/water permeable pore and neighboring TM1s cross and interact with each other in the inner leaflet of the bilayer through hydrophobic interactions between L19 and Val23 from one TM1 and G22 to G26 from the other. This configuration forms the most constricted hydrophobic part of the pore, called the “gate.” The outer TM2 helices flank the exterior of the channel and face the membrane; therefore, some TM2 amino acids are assumed to be responsible for sensing membrane tension (Yoshimura et al. 2004).

Conformational changes of MscL during channel opening have been investigated in electron paramagnetic resonance (EPR) and Föster resonance energy transfer (FRET) spectroscopic studies as well as molecular dynamics (MD) simulations (Gullingsrud et al. 2001; Elmore and Dougherty 2001; Perozo et al. 2002; Colombo et al. 2003; Gullingsrud and Schulten 2003, 2004; Meyer et al. 2006; Debret et al. 2008; Jeon and Voth 2008; Louhivuori et al. 2010; Sawada et al. 2012; Wang et al. 2014). Upon a membrane tension increase, TM1 and TM2 helices are dragged by lipids and gradually tilted down to the membrane plane, while the crossing portions (gate) between TM1s slide toward the radial direction, by which the gate expands (Gullingsrud et al. 2001, 2003; Corry et al. 2010; Sawada et al. 2012).

As the gate of MscL is narrow and hydrophobic, it is expected to exhibit hydrophobic gating; this property can be tunable by local changes in the size and hydrophilicity of the gate. Yoshimura et al. (1999, 2008) introduced a hydrophilic motif into the gate region, which induced a destabilization of the closed state and reduction of the gating threshold. They concluded that the hydrophobic nature of the gate probably stabilizes the closed state of MscL. This is supported by an experiment employing a mutant channel formed by the hydrophilic substitution of Gly22 with asparagine (G22N MscL), which spontaneously adopts an open substate even in the absence of membrane stretch.

The dewetting to wetting transition in the gate region is suggested to be relevant to the hydrophobic gating mechanism in ion channels (Beckstein et al. 2001; Beckstein and Sansom 2003). Anishkin et al. (2010) calculated the hydration energy for the MscL gate opening and discussed the importance of Gly22 and Gly26. Both amino acids are buried in the closed conformation but exposed to the lumen side in the open state, which would change the wetting characteristics of the pore (Anishkin et al. 2010). The hydrophobic nature of the MscL gate interrupts the continuous water column through the channel by forming a vapor plug between Leu19 and Val23. Two water–vapor interfaces are formed: one at the top (Val23) and the other at the bottom (Leu19) of the vapor plug. Thus, it is essential to understand the mechanism by which this vapor lock is broken, in other words, how increased membrane tension initiates gate wetting.

To acquire a detailed understanding of the wetting process of the MscL gate, we investigate the gate opening process, especially focusing on the relationship between gate wetting and conformational changes of the gate region. We visualize the wetting process during the course of gate expansion in MD simulations. To assess how the wetting of the gate influences gate expansion, we performed two types of simulations on MscL gating: one in which movements of water molecules are unrestrained (the normal model) and the other in which lateral movements of water molecules are restrained on the water–vapor interfaces at the periplasmic and cytoplasmic sides of the vapor bubble, plugging the pore of the closed gate. The latter model has never been tested, by which we can discuss the effect of hydration of the gate region in the initiation of gate opening triggered by increased membrane tension. We illustrate the crucial role of hydration of the backbone carbonyl oxygen atom of Leu19 that is exposed to the water-accessible space at the initial stage of gate expansion. Furthermore, we report that water wedging into the space between interacting TM1 helices facilitates further expansion of the gate toward channel opening.

## Methods

### System setup for simulation

**Fig. 1:**
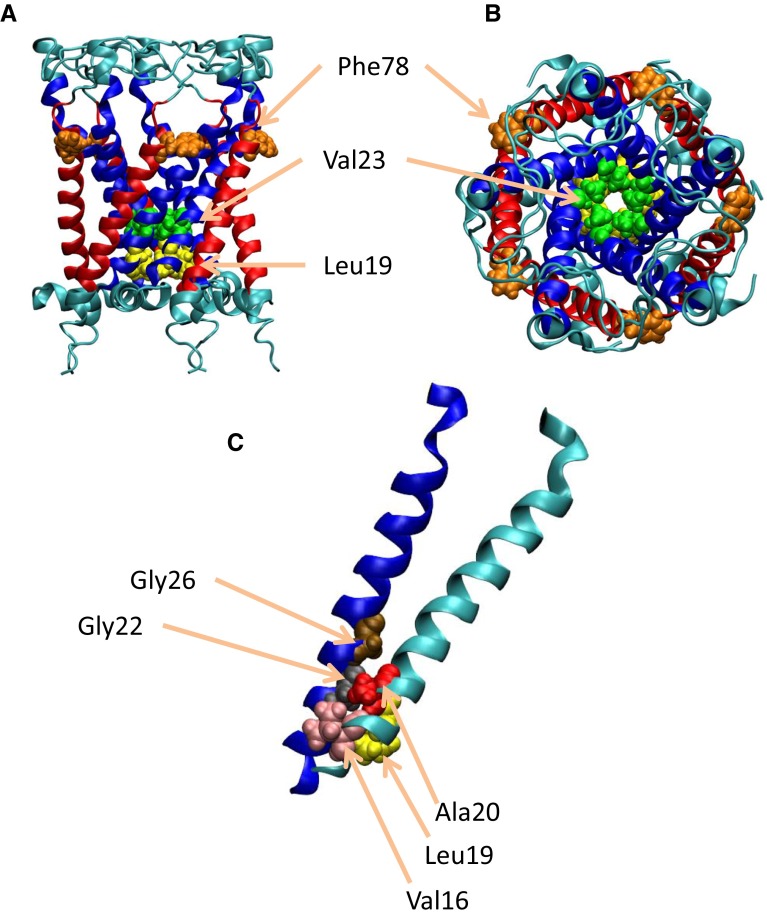
Three-dimensional structure of closed MscL. The *side* (**a**) and *top* (**b**) views of MscL from *Escherichia coli* are built on the MscL template from *Mycobacterium tuberculosis*. TM1 and TM2 helices are *colored blue* and *red*, respectively. **c** Conformation of the crossing formed by two neighboring TM1 helices (*colored blue* and *cyan*). Residues Val16, Leu19, Ala20, Gly22, Val23, Gly26, and Phe78 are depicted in *pink*, *yellow*, *red*, *black*, *green*, *brown*, and *orange* VDW representations, respectively. In the closed state, Gly22 in the TM1 helix of one subunit fits into the pocket formed by Val16, Leu19, and Ala20 in the left neighboring TM1. When the membrane tension increases, TM1s slide over each other, and the Gly22 residue is removed from the pocket and replaced with a Gly26 residue

**Fig. 2:**
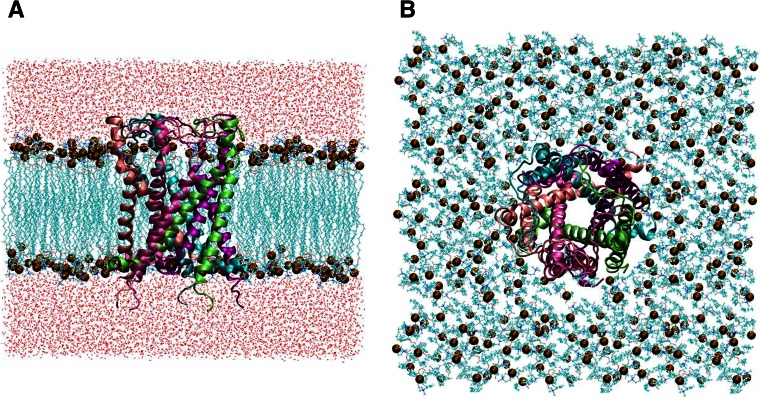
*Side* (**a**) and *top* (**b**) views of our simulation model comprising the *E*. *coli* MscL, POPC, and water molecules. MscL is shown in *ribbon view* with *different colors* for each subunit. The water molecules are shown in *red* (oxygen atoms) and *white* (hydrogen atoms). The *brown* atoms in the space-filling drawing are the phosphate atoms of individual lipid molecules

In this study, we first modeled MscL from *Escherichia coli* (*E*. *coli* MscL) in the closed state with S1 helices running parallel to the cytoplasmic membrane surface (Fig. 1). This model was based on the structure of MscL from *M*. *Tuberculosis* solved in 2007 (PDB code: 2OAR). The residues in the cytoplasmic region beyond Ala110 of the Eco-MscL, which have been suggested not to be essential for MscL gating, have been excised to reduce the total size of the system (Ajouz et al. 2000). The *E*. *coli* MscL model in a fully hydrated palmitoyl-oleoyl phosphatidylcholine (POPC) bilayer, which was utilized in our previous study, was solvated to place water molecules, as shown in Fig. 2, minimized over 10,000 steps with a fixed protein backbone, and then equilibrated for 50 ns under unrestrained conditions (351 lipids, 66 sodium and 71 chloride ions, approximately 23,000 water molecules, and approximately 125,000 atoms in total) (Grubmüller 1996; Sawada et al. 2012). After a 50-ns equilibration, opening simulations were performed with and without restraining lateral movements of the water molecules at the vapor–water interfaces of the periplasmic and cytoplasmic sides of the dewetted gate. In this study, the simulations with and without water restraints are denoted as *restrained water* and *unrestrained water*, respectively.

### Computational details

All MD simulations were performed by NAMD (ver. 2.9), utilizing the CHARMM27 force field and TIP3P water models (Darden et al. 1993; Reiling et al. 1996; MacKerell Jr. et al. 1998; Kalé et al. 1999). Both equilibrium and membrane stretching simulations were carried out in an NPT ensemble at 310 K and 1 atm. The particle mesh Ewald method was used for long-range electrostatic estimation, imposing a 12 Å cutoff for short-range electrostatic and van der Waals forces. Periodic boundary conditions (120 × 120 × 100 Å) were adopted. Visualization of states, molecular modifications, and analysis were conducted in visual molecular dynamics (VMD) using the embedded Tcl script language (Humphrey et al. 1996). When simulating the opening process, a negative pressure was generated at 150 dyn/cm along the lateral axis of the membrane, while a constant pressure of 1 bar was imposed in the *z* direction. Note that the timescale of the steered transition is much shorter than estimated in real experiments (approximately 10 μs), but it is sufficient for thermal relaxation of the sidechains along the opening path (Shapovalov and Lester 2004).

### Analysis

The interaction energies between MscL and the surrounding lipids and between the relevant amino acid residues (Leu19–Val23) and water were analyzed using VMD’s NAMDENERGY plug-in (Humphrey et al. 1996). Lipid and water molecules were selected from the area within the 12 Å cutoff distance from MscL (amino acid residues Leu19–Val23) in both restrained and unrestrained simulations. The MscL-lipid interaction energy was defined as the interaction energy difference between the closed (vapor-locked) state and the state in which the gate is expanded (hydrated). The energy was calculated as the sum of the interaction energy between each amino acid in MscL and the surrounding lipids. Here, the interaction energies include both electrostatic and van der Waals interactions.

The minimum pore radius of MscL was calculated by the HOLE program using a spherical probe (Smart et al. 1996) in the plane perpendicular to the pore axis at the amino acid (AA) 19’s position, which is presumably the most constricted portion of the pore. The distance between two amino acid residues was calculated from the three-dimensional coordinates of the backbone Cα atoms of both residues.

## Results

### Initiation of gate expansion as membrane tension increases in the normal MscL model

**Fig. 3:**
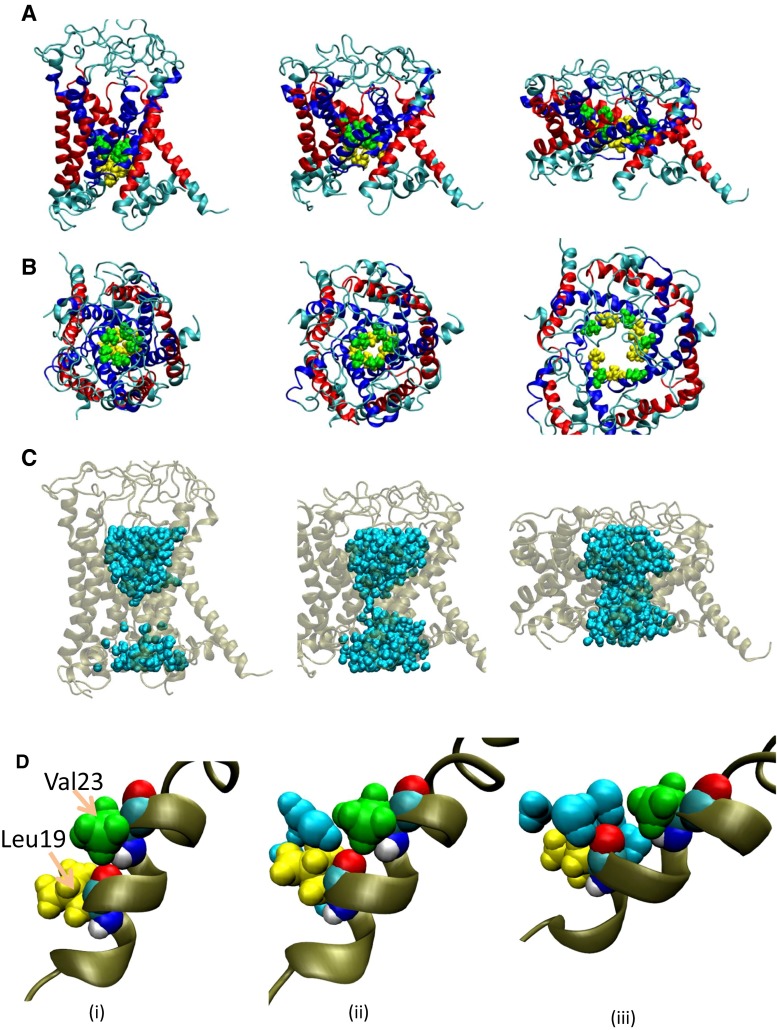
Snapshots of MscL structural changes as membrane tension increases in the unrestrained water (normal) simulation. **a** *Top views*, **b** corresponding *side views*, **c** water molecules around the gate, and **d** enlarged conformational changes at the gate of a TM1 helix, showing kinking and exposure of the backbone carbonyl oxygen atom of Leu19. *Columns* (*i*), (*ii*), and (*ii*) are taken at 0, 3, and 5 ns, respectively. Eco-MscL is shown in the *ribbon* representation; TM1 and TM2 helices are *colored blue* and *red*, respectively. The lipid and water molecules are excluded in *rows* **a** and **b** but water molecules are shown in *row* **c**

During the 50-ns equilibration period, MscL maintained its closed state in which the pore was lined with five TM1 helices. Immediately neighboring helices cross each other in the inner leaflet of the bilayer to form the most constricted pentagon-shaped structure called the gate (Fig. 1a, b). Gly22 in the TM1 helix of a subunit fits into a pocket formed by Val16, Leu19, and Ala20 from the immediately neighboring subunit. This hydrophobic interaction between neighboring TM1s stabilizes the closed state of the gate, thus being called the hydrophobic lock (Fig. 1c). Because of the small size of the closed gate and hydrophobic nature of its constituent amino acids (Leu19–Val23), no water was detected inside the gate, i.e., the gate was stably dehydrated in the closed state [Fig. 3c(i)].

**Fig. 4:**
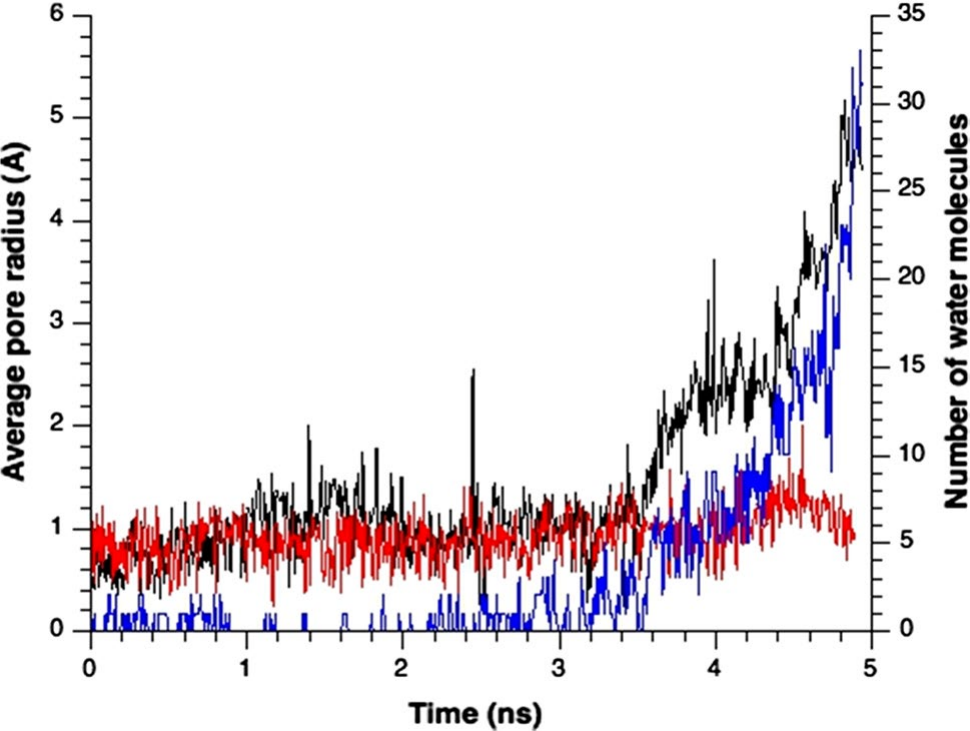
Time courses of changes in the pore radius and the number of water molecules at the gate of MscL in response to tension increase. *Black* and *red colored curves* denote changes in the pore radius at the most constricted portion of the pore with unrestrained and restrained water molecules, respectively. *Blue colored curve* depicts changes in the number of water molecules in the gate space formed between Leu19 and Val23 of TM1 helices in the unrestrained water model, while the number of water molecules was kept at zero (not shown) in the restrained water model. A tension increase was applied at time zero

When the membrane tension increased in the normal MscL model with unrestrained water, transmembrane helices (TM1 and TM2) were gradually tilted to the membrane plane, accompanied by outward sliding of the crossings between TM1 helices. By this mechanism, the gate expanded (Fig. 3a, b). Almost identical expanding behavior occurs in the MscL model with five bundled S1 helices in the cytoplasmic space (Chang et al. 1998); this model was adopted in our previous study (Sawada et al. 2012). As TM1 helices slide each other in the radial direction, the partner of the Leu19–Val23 pocket will shift from Gly22 to Gly26 (Fig. 1c). Further sliding leads to the most expanded state of the gate (radius = 5.4 Å in our simulations; Fig. 4). At approximately 3.5 ns of simulation, although the gate was still narrow with a radius of 1.7 Å [Figs. 3b(ii), 4], most of its hydrophobic surface had become hydrated [Fig. 3c(ii)]. Hydration was initiated by a single water molecule accessing the backbone carbonyl oxygen atom of Leu19 [Fig. 3d(ii)]. More precisely, the dragging force on TM1 arising from the tensile force exerted on Phe78 generated a kinking force near Leu19, which eventually broke the hydrogen bond between Leu19 and Val23. Consequently, the backbone carbonyl oxygen atom of Leu19 was exposed to the lumen of the pore, allowing interactions with water molecules, although the upper and lower vapor–water interfaces remained intact. Before complete merging of the upper and lower water phases, a single string of water molecules was formed across the constriction [Fig. 3c(ii)]. Then most of the hydrophobic surface of the gate came to be covered with water molecules, while the pore gradually expanded until the gate became completely wetted [Fig. 3c(iii)]. As shown in Fig. 4, the gate expanded slowly until 3.5 ns of simulation with few water molecules in the gate, but after 3.5 ns, it became considerably expanded, while the number of water molecules occupying the gate increased dramatically.

### Gating simulation of MscL with restrained water

**Fig. 5:**
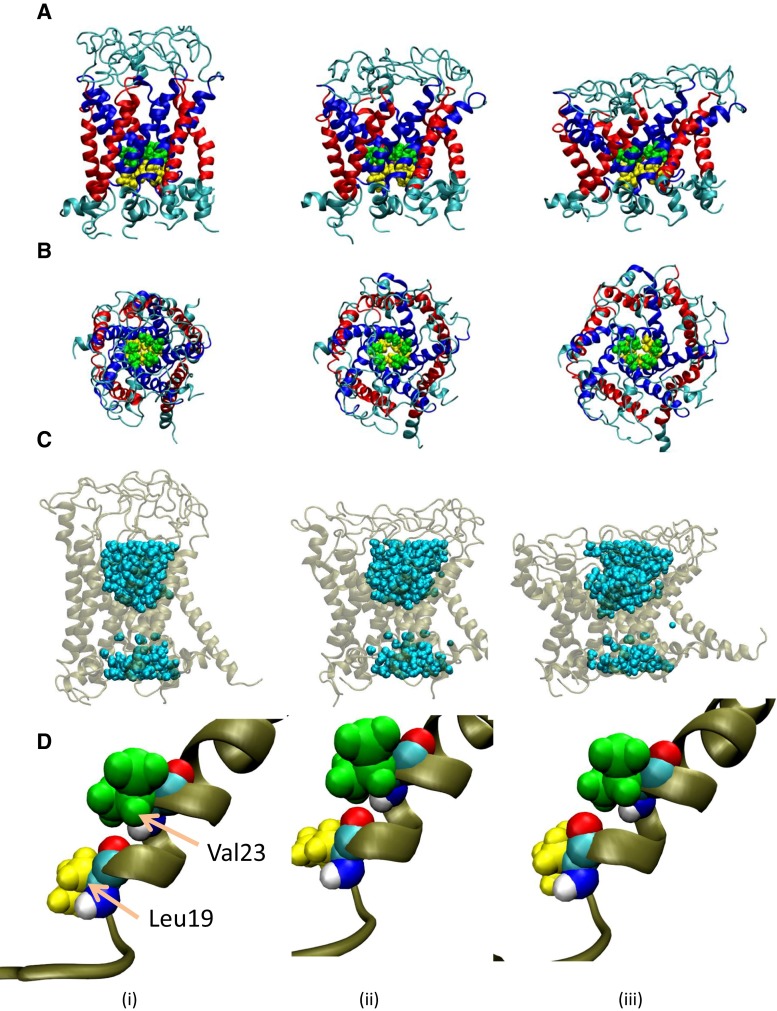
Snapshots of structural changes in MscL under increased tension in the restrained water simulation. **a** *Top views*, **b** corresponding *side views*, **c** water molecules around the gate, and **d** enlarged conformational changes at the gate of a TM1 helix, showing kinking and exposure of the backbone carbonyl oxygen atom of Leu19. *Columns* (*i*), (*ii*), and (*iii*) are taken 0, 3, and 5 ns, respectively. Eco-MscL is shown in the *ribbon* representation, with the TM1 and TM2 helices colored *blue* and *red*, respectively. The lipid and water molecules are excluded in *rows* **a** and **b** but are shown in *row* **c**

As described in the above subsection, breakage of the hydrogen bond between Leu19 and Val23 appears crucial to wetting and accelerated expansion of the gate with dramatic increases in the number of water molecules in the gate (Fig. 4). However, the relationship between the wetting and the accelerated gate expansion, whether these events are causally related, is not clear. Assuming that gate expansion is induced by water dynamics, we restrained the water movements around the gate, thereby eliminated their contribution to the gate expansion. After a 50-ns equilibration of the system, we restrained the lateral movement of water molecules forming the water–vapor interfaces and performed MscL opening simulations identical to the normal model. Figure 5 depicts conformational changes in the water-restrained MscL model induced by increased membrane tension. Early in the simulation time, the MscL protein behaved similarly to the normal MscL (with unrestrained water movements); the helices were tilted, and TM1 became kinked near Leu19, exposing the carbonyl oxygen of Leu19 to the pore lumen [Fig. 5d(ii, iii)]. However, no further expansion of the gate was observed (Fig. 5a, b), probably because the gate region was not allowed to interact with water (Figs. 4, 5). Comparing Figs. 3c and 5c, we infer that the opening mechanism of MscL is initiated by exposing the oxygen atom of Leu19 to the pore lumen, regardless of any interaction between water and the gate region.

### Effects of hydration on the energy and conformation of the gate

**Fig. 6:**
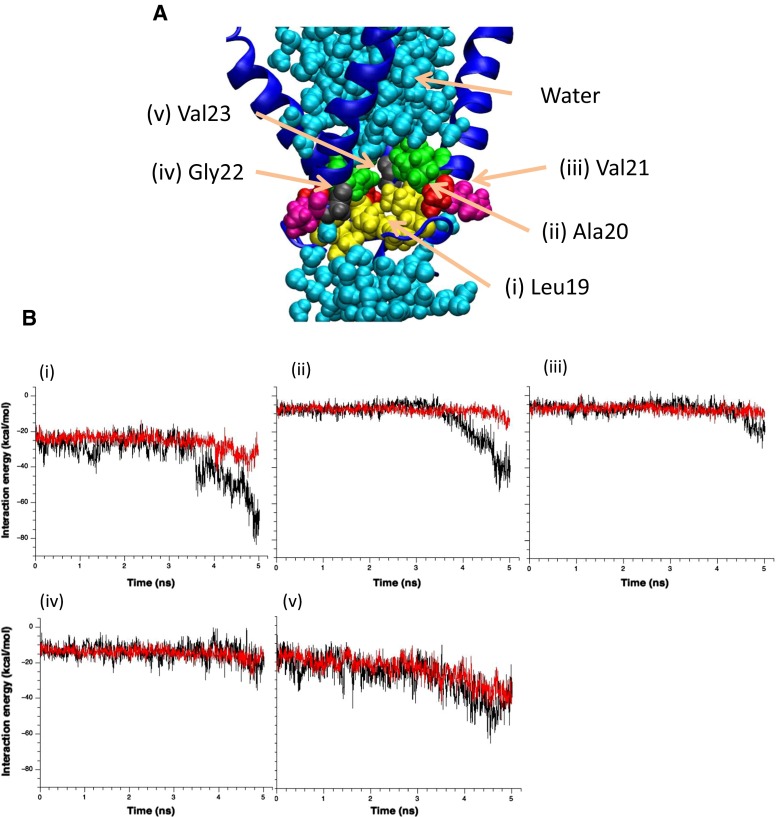
**a** Configuration of amino acids comprising the gate. Residues Leu19, Ala20, Val21, Gly22, Val23, and water molecules are depicted in *yellow*, *red*, *purple*, *black*, *green*, and *light blue*, respectively. **b** Temporal changes in the interaction energy between the above five amino acids and water induced by increased membrane tension. *Black* and *red lines* show the results of the unrestrained and restrained water models, respectively. The interaction energy consists of electrostatic and van der Waals interactions

As demonstrated in the above results, the backbone carbonyl atoms of Leu19s come to be exposed to the lumen of the pore regardless of the interaction with water. However, the extent of the gate expansion largely differed between the restrained and unrestrained models. To understand how hydration influences subsequent conformational changes of the gate, we calculated the interaction energy between the water molecules and amino acids (Leu19–Val23) constituting the gate in both models. Figure 6 shows the temporal changes in these interaction energies under increased tension. Almost all the interaction energies remained constant during the first 3 ns of the simulation. Thereafter, the Leu19–water and Ala20–water interaction energies gradually decreased in the unrestrained water model (reduced by 30–40 kcal/mol/5 subunits = 6–8 kcal/mol/subunit after 5 ns), whereas both interactions remained almost constant in the restrained water model [Fig. 6(i, ii)]. This result indicates that under increased tension, the value of the interaction energy after 3 ns results from the interaction between carbonyl oxygen of Leu19 and water, and hydration of the gate and the associated conformational changes are energetically favored over a dehydrated gate. Conversely, the closed state of MscL is stabilized by gate dehydration (vapor locking).

**Fig. 7:**
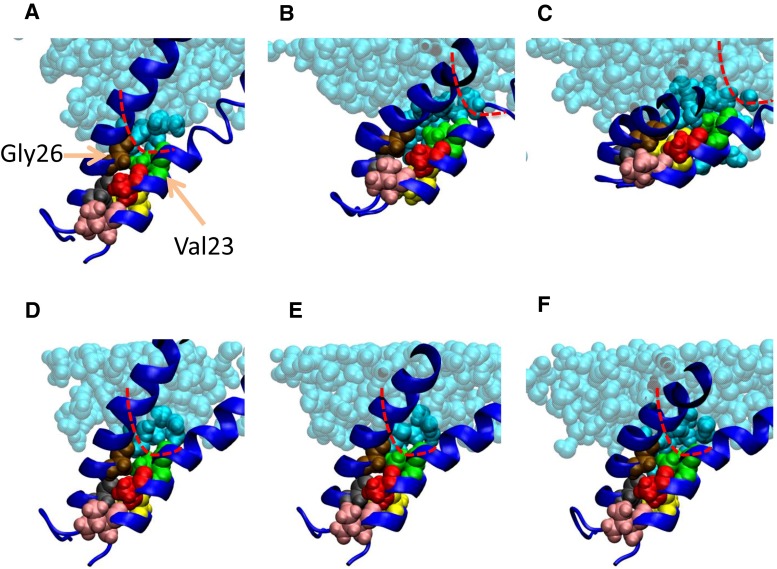
Snapshots of water movement and configuration changes of amino acids comprising the crossing portion formed by two neighboring TM1 helices when the membrane tension increases. **a**–**c** The configurations at 0, 3, and 4 ns, respectively, in the unrestrained water simulation. Corresponding snapshots in the restrained water simulation are presented in **d**–**f**. Residues Val16, Leu19, Ala20, Gly22, Val23, and Gly26 are shown in *pink*, *yellow*, *red*, *black*, *green*, and *brown* VDW representations, respectively. Water molecules (except those within 5 Å of Gly26) are depicted in the semitransparent *light blue* VDW representation. In all snapshots, the *red dashed line* indicates the boundary of the water–vapor interface on the periplasmic side of the pore at 0 ns simulation time

**Fig. 8:**
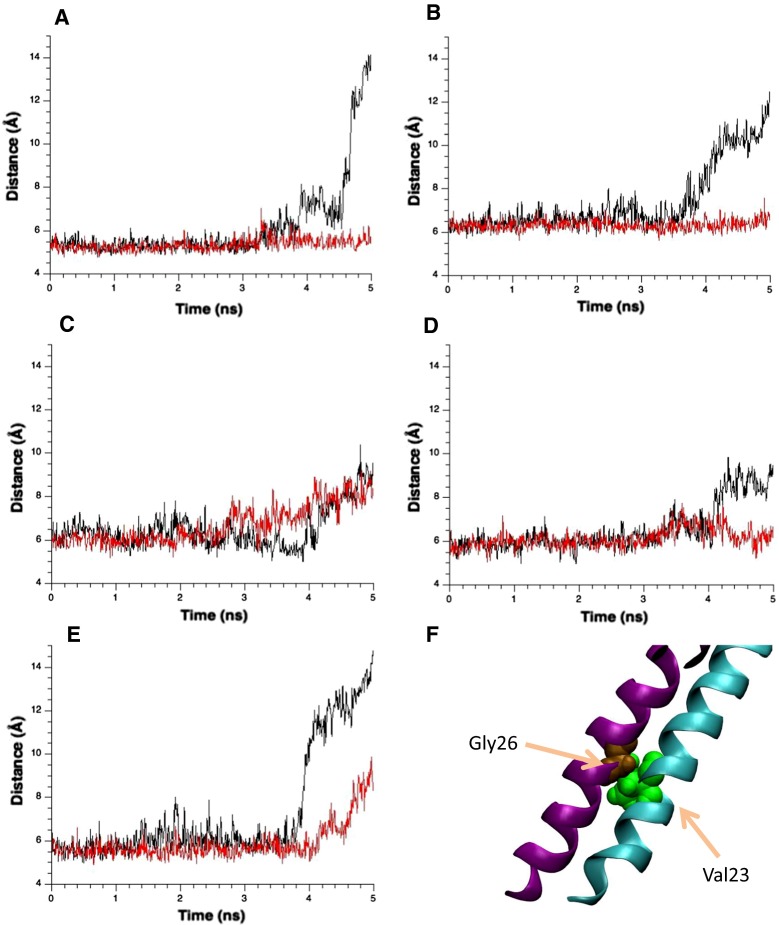
Temporal changes in the distance between Val23 in one TM1 and Gly26 of the neighboring TM1 with respect to five pairs (**a**–**e**). *Black* and *red lines* show the results from the unrestrained and restrained water models, respectively. The distance is defined as the distance between the backbone Cα atoms of amino acid residues. **f** Configuration of two neighboring TM1s in the *purple* and *cyan* ribbon representations. *Green* and *brown* residues denote Val23 and Gly26, respectively

Let us examine the mechanism of increased hydration of the gate after 3.5 ns, in other words, the relationship between hydration and apparently accelerated expansion of the gate, shown in Fig. 4. In the normal model, sliding of TM1 helices at the crossings was observed after an interaction between a water molecule and the exposed backbone oxygen atom of Leu19 occurred [Fig. 3d(ii)]. To understand the role of water molecules in the sliding of TM1 helices, i.e., accelerated gate expansion, we acquired snapshots of the water distribution around the crossing portions of TM1 helices throughout the simulation. A series of these snapshots is presented in Fig. 7a–c. At 3.5 ns, water molecules have wedged into the narrow gap between Val23 in one TM1 and Gly26 of its immediately neighboring TM1. Once this gap was fully occupied by water, Gly26 slipped past the gap to reach the pocket formed by Val16, Leu19, and Ala20. By contrast, the gate underwent minimal conformational change in the MscL model with restrained water movements (Fig. 7d–f). Figure 8 shows how the distance between Val23 on one TM1 and Gly26 from its immediate neighbor changes over time. Results are plotted for ten TM1 pairs: five from the normal model (black lines) and five from the restrained-water MscL model (red lines). In the normal model the distance between TM1s dramatically increased (Fig. 8a, b, e); in the restrained water model, it remained almost constant. This result confirms that entropy-driven water pressure induces conformational changes at the gate. We conclude that water molecules wedging between the helices act as a lubricator to decrease friction between the helices, which would promote the helix sliding toward further expansion of the gate. This may be the first mention on the role of helix sliding accelerated by water in the MscL opening. We may reasonably assume that water dynamics reduce the energy cost of the gate expansion toward channel opening.

## Discussion

Our previous simulation study showed that the channel opening of MscL upon membrane stretch is initiated by tilting down of the transmembrane inner and outer helices (TM1 and TM2, respectively) toward the membrane plane followed by gate expansion (Sawada et al. 2012). The present study demonstrates that restraining the water movement at the water–vapor interfaces severely inhibits the gate expansion but does not influence the helix tilting (Fig. 5a). Based on this, we propose that the initial phase of MscL gating comprises two steps: (1) tilting of the transmembrane helices and (2) gate expansion. We discuss here the likely conformational changes at the MscL gate, particularly focusing on the interplay between the gate and water molecules in atomic detail.

In the first gating step (tilting of the transmembrane helices), membrane tension is first sensed by the Phe78 residue located at the periplasmic lipid–water interface. In response to increased membrane tension, the lipids around Phe78 drag the TM2 and TM1 helices and gradually tilt them toward the membrane plane (Sawada et al. 2012). This dragging force eventually kinks TM1 between Leu19 and Val 23. The structure just below the kink position (Leu19) acts as a fixed point of a lever arm constituted by TM1 and TM2. Once the kink has broken the hydrogen bond between the oxygen atom of Leu19 and the amide hydrogen atom of Val23, the carbonyl oxygen atom becomes exposed. The dragging force is probably transmitted to the inner TM1 helix via a periplasmic loop and/or a strong salt bridge between Lys31 in TM1 and Asp84 in the immediately neighboring TM2 helix. Alpha helices are usually mechanically rigid, except at their glycine and proline residues. In our simulations, no kink appeared at the Gly22 or Gly26 of TM1 helices, but the position just above Leu19 was slightly kinked by increased membrane tension. As shown in Fig. 1c, Leu19 resides in the pocket of TM1 and is tightly packed with Gly22 in the closed MscL. Under increased tension, TM1 is tilted down toward the membrane plane with a slight kink at Leu19 (Fig. 3d). The neighboring TM1 helices are bundled around the pocket and can be regarded as a tilting arm lever pivoting around Leu19. If this is true, the mechanical stress generated in the TM1 helix should be concentrated there. However, why Leu19 is the pivotal residue remains uncertain. One possible mechanism is the small interaction energy between the backbone carbonyl oxygen atom of Leu19 and the backbone amide hydrogen atom of Val23 (−96 kcal/mol) relative to those of other nearby hydrogen bonds: Val17 and Val21 (approximately −108 kcal/mol), Ala20 and Ile24 (approximately −108 kcal/mol), and Val21 and Ile25 (approximately −120 kcal/mol). Only the Asp18–Gly22 pair possesses a smaller hydrogen bond energy (approximately −88 kcal/mol), but this energy gradually decreased to approximately −115 kcal/mol during the course of channel opening, whereas other hydrogen bond energies were unaltered.

Next, let us consider the energetic feasibility of the breakage of the hydrogen bond under increased membrane tension. The tension-dependent tilting down of the transmembrane helices must result from the work done at the tension-sensing sites of MscL. Three amino acid residues, Phe78, Ile79, and Phe83, located at the periplasmic side of the TM2 helices, have been identified as candidate tension sites by single-site mutagenesis (Yoshimura et al. 2004). The approximate work done by protein–lipid interactions has been estimated at 120 kcal/mol/MscL, assuming that each amino acid residue involved in gate opening receives a pulling force of 70 pN (Gullingsrud and Schulten 2003). In our simulations, the above candidate amino acid residues were dragged by approximately 8 Å before the hydrogen bond was broken. Given that the total energy necessary to break the hydrogen bonds of all Leu19s in an MscL is approximately −96 kcal/mol, the energy calculated from the lipid–protein interactions (120 kcal/mol) is sufficiently large to break the hydrogen bond between Leu19 and Val23, assuming that the energy conversion efficiency from tension sensing to helix kinking is 100 %. In addition, since the membrane tension was approximately ten times higher in our simulation than in the usual experiments to activate MscL, the magnitude of the force loaded on the tension-sensing sites in our MscL models should far exceed 70 pN. Furthermore, as shown in Fig. 5d, exposure of the backbone carbonyl oxygen atom of Leu19 occurred even in the restrained water simulation. Thus, we suggest that exposure of the backbone carbonyl oxygen atom of Leu19 is a causal process in gate expansion, enabling a pioneering water molecule to access the exposed oxygen atom and initiate the dewetting to wetting transition of the gate region.

One may suspect that the breakage of the hydrogen bond between Leu19 and Val23 was just caused by an extremely high value of applied membrane tension (150 dyn/cm). Although we cannot rule out this possibility, we discuss here that this may not be the case in the present study. We generated membrane tension by decreasing the lateral pressure only in the lipids; MscL is not directly subjected to the generated pulling force. According to our previous study (Sawada et al. 2012), the major pulling force to MscL is transmitted via Phe78 and reaches the gate through an unanalyzed complicated viscoelastic process. This process in general can be analyzed by using the Maxwell or Voigt model, or their combination; a Voigt model composed of an elastic spring and a dashpot in parallel under constant force may be appropriate in the present case. Structural changes of MscL upon tension increase correspond to the creeping in the Voigt model, where only a very small amount of creeping can be observed in the initial phase of the mechanical response because most of the applied force is balanced by friction of the dashpot. According to a calculation using a coarse grain model of MscL, it takes over 1 μs to attain full opening of MscL at 30 dyn/cm (Deplazes et al. 2012). Therefore, several ns of MD simulation would reflect only a very initial transient creeping, and the amplitude of applied force on the protein (MscL) creeping during such a short period is exerted mainly in the time course of creeping. We have actually challenged MscL opening simulations with membrane tension higher (250 dyn/cm) or lower (75 dyn/cm) than 150 dyn/cm. The results indicate that the structural changes of MscL in these conditions are essentially the same (not shown) as those in 150 dyn/cm simulation, including an exposure of the carbonyl oxygen of Leu19. The only difference between the results with different forces was the time course, i.e., the time needed for gate expansion to a certain level gets shorter with the strength of applied forces, which is consistent with the expectation from the Voigt model.

As described in the text, exposure of the oxygen atom of Leu19 was observed in both the water-restrained and non-restrained (normal) models, yet no further gate expansion was undergone in the former model, while gate expansion proceeded in the latter model. Such distinct structural responses between the models suggest that our applied force (150 dyn/cm) is not an arrogant one that induces nonspecific destructive structural changes of MscL. Collectively, the exposure of the oxygen of Leu19 in our study might not occur as a result of excessive membrane tension, but rather one of the essential conformational changes of MscL toward channel opening upon membrane stretch.

To estimate the energy contribution of the dewetting to wetting transition at the gate, we calculated the hydration energy of the gate constituents (Leu19–Val23). The approximate energy difference was 30–40 kcal/mol (Fig. 8), which is consistent with that of Anishkin et al. (2010) who assumed that the hydration area was increased by 20 nm^2^ and thereby calculated the gate hydration energy at −30 kcal/mol. Collectively, these results strongly support the idea that gate wetting is energetically beneficial for MscL under increased membrane tension even though the local hydration of the hydrophobic gate is energetically unfavorable.

During the gate expansion that leads to channel opening, sliding between the neighboring TM1 helices in the crossing region is roughened by mechanical and chemical interactions between the residues (Val16, Leu19, and Ala20) in TM1 and those in its neighboring TM1 (Gly22 and Gly26). In our previous study, we calculated a kind of activation energy (approximately 25 kcal/mol) to carry out the sliding toward channel opening (Sawada et al. 2012). In the “restrained water” model, however, no obvious TM1 sliding, and therefore no gate expansion, was observed (Fig. 7d–f). This suggests a strong association between the TM1 sliding and gate hydration. At the water–vapor interface on the cytoplasmic side, Val23 closely contacts Gly26 of the immediately neighboring subunit in the closed state. As the gate opens, Gly26 is bypassed because the Val23–Gly26 gap becomes occupied by water molecules. In the normal MscL model, the distance between Gly26 and Val23 is widened in at least three of the five subunits at 3 ns from the onset of the tension increase. On the other hand, this distance remains nearly constant in the restrained water simulation (Fig. 8a, b, e). It appears that the sliding of TM1 helices is assisted by the penetration of the water molecules around the vapor–water interface into the gate (TM1 crossing region), driven by the entropic pressure of periplasmic bulk water. By facilitating the helix sliding, water appears to play an essential role in the conformational changes leading to further expansion of the gate and the following increased number of water molecules into the gate region (Fig. 4). Once gate hydration becomes energetically favorable as estimated above, MscL can be fully opened by further conformational changes at the gate.

Finally, we want to touch on the apparently contradictory result showing an opening behavior of MscL using an MD model without water (Gullingsrud et al. 2001). As detailed data on this result are not provided in their paper, we can only point out the possible causes that produce the apparent discrepancy. First, their method of applying forces to MscL is obviously different from ours. They applied lateral pulling force directly to the main chains of MscL facing the lipids. An important point is that the force is uniform with respect to the transmembrane axis, which means not only the portion around the water–lipid interface, which we believe is a tension-sensing site of MscL (Yoshimura et al. 2004; Sawada et al. 2012), but also the region around the gate located at the membrane core is subjected to pulling force. As the main chain around the MscL gate is rather subjected to an increased pressure from the membrane core upon membrane stretch, their method of force application is just opposite to the prospective physiological situation. Therefore, we suspect that their result showing the seeming opening of the MscL model without a membrane and water may be mainly caused by such an unphysiological force application. It may be trivial, but their MscL model is based on the structure of *M*. *tuberculosis*, while ours is based on *E*. *coli*, which might be one of the causes of the different results between their study and ours. Furthermore, MD simulations without any water may not be realistic because protein dynamics generally undergo a tight association with water dynamics. Our model was constructed to depict the effect of gate hydration with minimum perturbation of the protein dynamics, and the model successfully demonstrates the critical role of water in the gate opening of MscL.

## Conclusions

We performed MD simulations of the mechanogating process in the bacterial mechanosensitive channel MscL under two conditions of water dynamics. In one condition, water molecules forming the vapor–water interfaces at the gate were unrestrained (the normal version of the model); in the other condition, their lateral motions were restrained. In both conditions, the transmembrane helices were tilted in response to increased membrane tension, which induced breakage of the Leu19–Val23 hydrogen bond, resulting in an exposure of the carbonyl oxygen of Leu19 on the TM1 backbone. In the normal model, the pioneering water molecule stably interacted with the exposed oxygen, allowing further penetration of water molecules into the gate region. Some of these water molecules wedged into the gap space at the crossing portion between the immediately neighboring TM1s and reduced the friction of the helix sliding that leads to gate expansion. Consequently, the gate further expanded and admitted water chains across the gate region, followed by dramatic increases in the number of water molecules penetrating into the gate. By contrast, the water-restrained model showed a similar exposure of the carbonyl oxygen of Leu19, but no further conformational change was observed. Thus, the initial phase of gate expansion was separable into two steps. The first step, a water-independent step, is purely mechanical; the Leu19–Val23 hydrogen bond is broken to expose the carbonyl oxygen atom of Leu19. The second (water-dependent) step is a wetting process that weakens the hydrophobic lock, committing the gate expansion toward channel opening.
